# Importance of Floating Chondrons in Cartilage Tissue Engineering

**Published:** 2017-01

**Authors:** Hajar Shafaei, Hajar Bagernezhad, Hassan Bagernajad

**Affiliations:** 1 Department of Anatomical Sciences, Faculty of Medicine, Tabriz University of Medical Sciences, Tabriz, Iran;; 2 Radiology Department, Sina Hospital, Tabriz University of Medical Sciences, Tabriz, Iran

**Keywords:** Cartilage tissue engineering, Chondron, Condrocyte, Dedifferentiation, PCM

## Abstract

**BACKGROUND:**

Dedifferentiation of chondrocytes remains a major problem for cartilage tissue engineering. Chondrocytes loss differentiated phenotype in in vitro culture that is undesired for repair strategies. The chondrocyte is surrounded by a pericellular matrix (PCM), together forming the chondron. PCM has a positive effect on the maintenance of chondrocyte phenotype during culture in comparison to uncovered chondrocyte. Studies suggest that the PCM influence on functional properties of the chondrocytes. However there is no study to show gene expression phenotype differences between round chondron and fibroblastic chondrocytes. We aimed to investigate the effect of pericellular matrix in maintaining of chondrogenic gene expression to solve dedifferentiation problem of chondrocyte.

**METHODS:**

In this study enzymatically isolated chondrons were cultured for 7 days. Morphology of chondrons were assessed by microscopic examination. Chondrogenic gene expression of Sox9, aggrecan (AGG), cartilage oligomeric matrix protein (COMP), Link protein and chondro-osteogenic gene expression (Runx2, Col1, Col 10 and MMP13) of attached and float chondrons were assessed by real time RT PCR.

**RESULTS:**

Microscopic observation showed that round shape of chondron observed at day 7 in floating chondrocytes. Gene expression results showed that attached chondrons significantly dedifferentiated by low gene expression of Sox9 and COMP and high MMP13 versus floating cells.

**CONCLUSION:**

Our results showed that PCM of chondrocyte could restore differentiated state of chondrocytes at day 7. Using unattached form of chondron in cartilage tissue PCM in maintenance of chondrogenic gene expression engineering could be a novel method to solve dedifferentiation problem of chondrocyte.

## INTRODUCTION

Articular cartilage tissue shows a limited capacity for self-repair.^1^ In cartilage tissue engineering, it is often used isolated chondrocytes and their expansion is necessary to obtain sufficient cells.^2^ However in expansion culture period, chondrocytes dedifferentiate and lose their specific chondrocytic phenotype. They start producing type I instead of type II collagen and also decrease proteoglycan synthesis.^3^ Studies have been shown that constructed cartilage tissue from primary chondrocytes cultures resemble natural cartilage than those of higher passaged chondrocytes.^4^ The maintenance of the differentiated chondrocyte phenotype in cartilage tissue engineering is essential challenge.^5^^-^^7^

In cartilage, chondrocyte with its pericellular matrix (PCM) named chondron.^8^ The PCM has a defined molecular structure and unique mechanical properties that support the chondrocyte and plays a crucial role in homeostasis of cartilage tissue.^9^ Chondrocytes communicate with extracelluar matrix via PCM. During expansion of chondrocytes, considerable changes occur at various levels of chondrocyte synthesis including the extracelluar matrix, cell surface receptors and cytoskeletal proteins.^10^ Sequential passages of articular cartilage resulted in reduction of type 2 collagen and aggrecan as chondrocyte differentiation markers and increase in type 1 collagen and MMP13 as classic dedifferentiation marker.^11^ The mechanisms of dedifferentiation is not clear, however the results of studies suggest that the effect of culture surface on cell morphology is a consequence of the process of interaction between the extracellular matrix (ECM) proteins.^12^ Therefore this study designed to investigate the first stage of dedifferentiation of chondrocyte when chondrocytes have round morphology in PCM and fibroblastic form without PCM.

## MATERIALS AND METHODS

Human articular cartilage samples (surgical waste) were harvested during total knee arthroplasty with the informed consent. The tissues were dissected and minced. Cartilage particles were digested with 0.3% (w/v) dispase (Gibco,USA) plus 0.2% (w/v) collagenase in phosphate buffered saline (PBS; Gibco, USA) for 5 h as previously described in shaking water bath at 37˚C.^13^ For neutralizing enzyme, the same volume of medium DMEM (Gibco, USA) containing 10% FBS (sigma, USA) and 1% penicillin/streptomycine (Gibco, USA) were added. The cells were filtered through a 70 mm cell strainer (BD Biosciences, San Diego, CA, USA) and washed. 

The chondrons were cultured in DMEM supplemented with 50µg/ml ascorbic acid, 10% FBS, 100 U/ml of penicillin, 100 mg/ml of streptomycin, 2.5 mg/ml of amphotericin B. For neutralizing enzyme, the same volume of medium DMEM (Gibco, USA) containing 10% FBS (Sigma, USA) and 1% penicillin/streptomycine (Gibco, USA) were added. The obtained cell suspension were washed by centrifuging in 1600 rpm for 10 min and were added medium containing DMEM, 10% FBS, 1% penicillin / streptomycin and ascorbic acid (0.05 mg/ml). Then the cell suspension transferred to flasks and were cultured in incubator 37 ˚C, 88% humidity and 5% CO2 for 2-6 weeks. Culture medium were replaced every 3-4 days. After 80% confluency, the cells were passaged.

After experimental period, the medium was removed, and the cells were washed with phosphate-buffered saline (PBS), fixed with 4% formaldehyde for 10 min and incubated with 0.1% alcian blue (Sigma, USA) in 3% acetic acid (pH 2.0) for metachromatic proteoglycan staining for two 2 hours.

RNA isolation was performed using TRIzol ® reagent (Invitrogen, Carlsbad, CA, USA). cDNA synthesis was performed using Masterscript Vilo®. Real-time PCR reactions were performed using the SYBRGreen reaction kit. cDNA (approx. 5 ng) was used in 20 ml PCR mix (LightCycler DNA Master Fast start ^plus^ Kit, Roche Diagnostics) containing a final concentration of 0.5 pmol of primers (Table 1). 

**Table 1:** **:** Real-time RT PCR primers used in this study

**Primer**	**Primer sequence**	**Product size (Bp)**
18SR	5’- GTAACCCGTTGAACCCCATT- 3’	153
18SF	5’- CCATCCAATCGGTAGTAGCG 3’	
Col10F	5’-CACTACCCAACACCAAGACA-3’	225
Col10R	5’- CTGGTTTCCCTACAGCTGAT-3’	
Sox9F	5’-CCCAACGCCATCTTCAAGG-3’	242
Sox9R	5’-CTGCTCAGCTCGCCGATGT-3’	
AGGF	5’-CAACTACCCGGCCATCC-3’	160
AGGR	5’-GATGGCTCTGTAATGGAACAC-3’	
Col1F	5’-AAGCCGAATTCCTGGTCT-3’	195
Col1R	5’- TCCAACGAGATCGAGATCC-3’	
Runx2F	5′ -ATGCTTCATTCGCCTCAC-3′	156
Runx2R	5′- ACTGCTTGCAGCCTTAAAT-3’	
MMP13F	5’-GGAGCATGGCGACTTCTAC-3’	208
MMP13R	5’-GAGTGCTCCAGGGTCCTT-3’	
ALPF	5’-CCCACAATGTGGACTACCT-3’	143
ALPR	5’-GAAGCCTTTGGGGTTCTTC-3’	
COMPF	5-’ AACGCGGCGCTGCAGGAC-3’	246
COMPR	5’–CGAGCCGTTGCCCGTGAAG-3’	

Bp: Base pair; F: Forward; R: Reverse 18s as housekeeping gene; Col10: Collagen type 10; Sox9: SRY (sex determining region Y)-box 9; AGG: Aggrecan; Col1: Collagen type I; Runx2: Runt-related transcription factor-2; MMP13: Matrix metalloproteinase-13; ALP: Alkaline phosphatase; COMP: Cartilage oligomeric matrix protein; Link P: Link protein

Relative target gene expression of main chondrogenic markers (SOX9, Col2A, aggrecan (AGG), cartilage oligomeric matrix protein (COMP), and Link protein) and mixed osteochondrogenic markers such as Runx2, collagen type 10 (Col10), collagen type I (Col1), and matrix metalloproteinase-13 (MMP13) calculated to housekeeping gene expression (18s) by Light Cycler software version 4. Crossing points were assessed, and plotted versus a serial dilution of individual gene standards using Fit Points method. PCR efficiency was calculated, and only data with efficiencies ranging from 1.85-2.0 were used.

Data were obtained from three independent donors. For statistical analysis, the data were presented as means±SEM .Significances were tested by one way ANOVA in Graph Pad prism program. Differences were considered significant if *p*<0.05.

## RESULTS

**Fig. 1:**
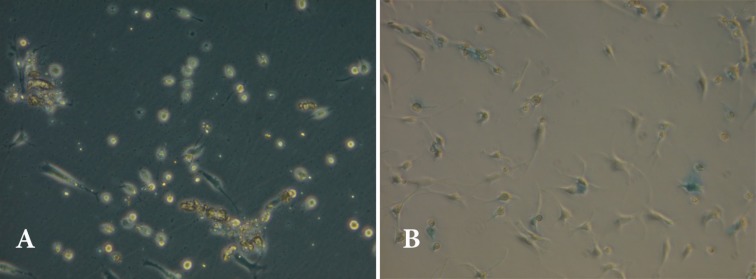
Micrographs showing chondrons at day 7. A) Attached and floated chondrons by inverted microscope. B) Alcian blue staining showing proteoglycan around round cells (arrow) and attached cells

In this study, dedifferentiation of chondrocyte and loss of PCM were evaluated by microscope at day 7. Gene expression of Sox9, AGG ،Col2A, COMP, Link P, MMP13, Runx2, Col1, Col10 in float and attached chondrons were investigated at day 7. Microscopic observation of this study showed that float chondrons maintain their round morphology and PCM (Figure 1A). Alcian blue staining confirmed proteoglycan around chondrons but not around any proteoglycan around fibroblastic chondrocytes (Figure 1B). 

**Fig. 2:**
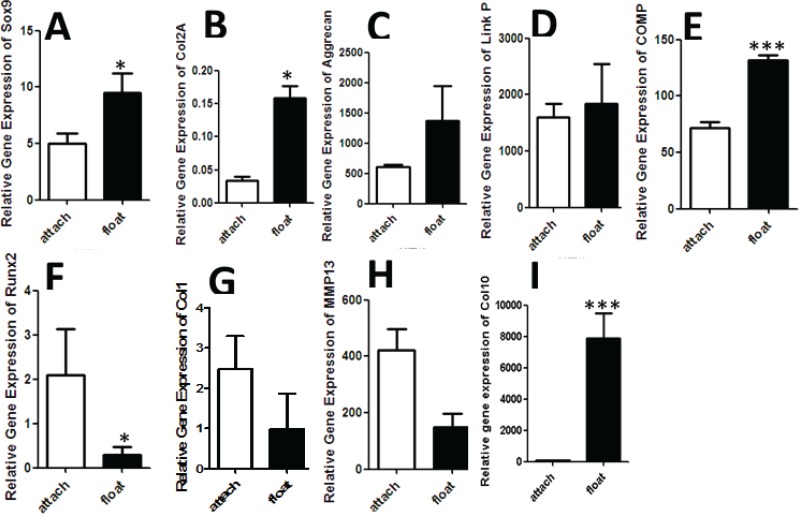
Chondrogenic gene expression Sox9, AGG ،Col2A, COMP, Link P, MMP13, Runx2, Col1, ALP, Col10 in attached and float chondrons at day 7. A) Sox9 gene expression in attached and float chondrons at day 7. High gene expression of Sox9 in float chondrons versus attached chondrons (*p*<0.05). (B) Col2A gene expression in attached and float chondrons at day 7C). AGG gene expression in attached and float chondrons at day 7. D) Link P gene expression in attached and float chondrons at day 7. E) COMP gene expression in attached and float chondrons at day 7.

Attached chondrons in comparison to float chondrons dedifferentiated and expressed low Sox9 and Col2A genes at day 7 (*p*<0.05) (Figure 2.A and B). The expression of aggrecan and Link protein genes was low in attached chondrons than float chondrons (Figure 2.C and D). Also COMP gene expression was significantly high in float chondrons (*p*<0.0005) (Figure 2.E) The expression of Runx2 and Col1 genes was high in attached chondrons than float chondrons (Figure 2 F and G). MMP13 gene expression was significantly high in attached chondrons at day 7 (*p*<0.05) (Figure 2 H). Our results showed that the expression of Col10 was significantly (*p*<0.0005) low in attached chondrons (Figure 2 I). 

Decreasing of COMP gene expression in attached chondrons were significant versus float chondrons (*p*<0.0005). F) Runx2 gene expression in attached and float chondrons at day 7. Increasing of Runx2 gene expression in attached chondrons were significant versus float chondrons (*p*<0.05). G). Col1 gene expression in attached and float chondrons at day 7. Increasing of Col1 gene expression in attached chondrons were significant versus float chondrons (*p*<0.05). H) Col10 gene expression in attached and float chondrons at day 7.

Decreasing of Col10 gene expression in attached chondrons were significant versus float chondrons (*p*<0.05). Values are mean ± SEM, n=3. Sox9, SRY (sex determining region Y)-box 9; Col2A, collagen type 2A; AGG, aggrecan; COMP, cartilage oligomeric matrix protein; Link P, Link protein; Col1, collagen type I; Runx2, runt-related transcription factor-2; MMP13, matrix metalloproteinase-13; ALP, alkaline phosphatase; Col10, collagen type 10.

## DISCUSSION

In cartilage tissue engineering, proliferation of chondrocyte, cell death and chondrocyte dedifferentiation are major challenges.^14^ A rapid increase in mRNA expression of type 1 collagen occurs whereas a significant decrease of type 2 collagen and Sox 9 was observed in chondrocytes through the successive passages.^15^ After transplantation of such cells to cartilage tissue, fibrocartilage forms in defect site. One strategy for solving this problem is preserving of matrix of cell which is called PCM. In fact chondron is chondrocyte with PCM. 

The results of this study showed that PCM maintenance preserves chondrocyte phenotype up to day 7. After losing of PCM, chondrocytes attach to plastic surface and dedifferentiation starts. Therefore if PCM preserves during chondrocyte isolation, hyaline cartilage formation in defect site increases and articular cartilage repair improves. Previous studies have been shown that for maintaining of PCM, mechanical isolation methods of chondron are more effective than enzymatic methods.^16^ In this study, enzymatic method has been used that could affect attachment time of chondron on surface and gene expression. However it has been attended that enzymatic isolation method of chondron harvest more cell with high viability and this method is more feasible than mechanic method as well.^17^

Cultured chondrocytes on surfaces with loose attachment in comparison with plastic surfaces expressed more collagen type 2, aggrecan and link protein and less collagen type 1.^18^ The possible explanation of these results is associated with integrin and cadherins and attachment of chondrocyte to cell surface mediated by high expressoion of cadherin^19^ and fibronectin.^12^ It seems that cell membrane proteins of chondrons remain intact in PCM and communicate with extracellular matrix proteins and preserves dedifferentiation of chondrocyte. 

In this study, high collagen type2A and collagen type 10 expression is probably due to our osteoarthritis samlples.^20^ Low collgen type 10 in attached chondrocytes indicates that cells tend to transform fibroblastic phenotype than hypertrophic chondrocyte. On the other hand in PCM quality of osteoarthritic chondrocytes, is different from normal chondrocyte.^21^ Therefore further evaluations, it is needed to show our results with human normal chondrons but there is limitation for harvesting of normal articular cartilage.

The passages of chondrocyte cultures related to dedifferentiation which mostly occurs in higher passages,^22^ however our results show that dedifferentiation starts earlier in primary culture. We showed that Sox9 is master gene of chondrogenesis^23^ and decreasing of this gene has been shown at day 7. Our results showed that PCM of chondrocyte could restore differentiated state of chondrocyte in day 7. Using unattached form of chondron in cartilage tissue engineering could be a novel method to solve dedifferentiation problem of chondrocyte.
